# Response of Terrestrial Net Primary Production to Quadrupled CO_2_ Forcing: A Comparison between the CAS-ESM2 and CMIP6 Models

**DOI:** 10.3390/biology11121693

**Published:** 2022-11-24

**Authors:** Jiawen Zhu, Xiaodong Zeng, Xiaofei Gao, He Zhang

**Affiliations:** 1 International Center for Climate and Environment Sciences, Institute of Atmospheric Physics, Chinese Academy of Sciences, Beijing 100029, China; 2 Collaborative Innovation Center on Forecast and Evaluation of Meteorological Disasters (CIC-FEMD), Nanjing University of Information Science & Technology, Nanjing 210044, China; 3 University of Chinese Academy of Sciences, Beijing 100049, China

**Keywords:** terrestrial net primary production, quadrupled CO_2_, CO_2_ fertilization effects, climatic effects, CAS-ESM2, CMIP6

## Abstract

**Simple Summary:**

Terrestrial ecosystems, a key part of the Earth system, can absorb about 31% of CO_2_ emissions generated by human activities, which slows increasing atmospheric CO_2_ concentrations and global climate warming. The ways in which terrestrial net primary production responds to elevated CO_2_ and associated climate change is a key topic of climate sciences. This study estimated the responses of net primary production to quadrupled CO_2_ using 23 Earth system models, and analyzed the underlying causes of uncertainties among the models. The results showed that all of the models projected positive responses of net primary production, with an averaged magnitude of 44.78 PgC year^−1^ and an uncertainty of ±20.93 PgC year^−1^. The uncertainty came from many sources, and this study emphasizes the role of different projections in CO_2_-induced climatic anomalies and different climate sensitivities. These results provide a reliable estimation of global net primary production response to quadrupled CO_2_, and point out that more understanding and improvements are needed for Earth system models to represent both physical climatic processes and the terrestrial carbon cycle.

**Abstract:**

Terrestrial net primary production (NPP) is a key carbon flux that changes with rising atmospheric CO_2_ and CO_2_-induced climate change. Earth system models are commonly used to investigate these NPP changes because of their fundamentally trustworthy ability to simulate physical climate systems and terrestrial biogeochemical processes. However, many uncertainties remain in projecting NPP responses, due to their complex processes and divergent model characteristics. This study estimated NPP responses to elevated CO_2_ and CO_2_-induced climate change using the Chinese Academy of Sciences Earth System Model version 2 (CAS-ESM2), as well as 22 CMIP6 models. Based on CMIP6 pre-industrial and abruptly quadrupled CO_2_ experiments, the analysis focused on a comparison of the CAS-ESM2 with the multi-model ensemble (MME), and on a detection of underlying causes of their differences. We found that all of the models showed an overall enhancement in NPP, and that CAS-ESM2 projected a slightly weaker NPP enhancement than MME. This weaker NPP enhancement was the net result of much weaker NPP enhancement over the tropics, and a little stronger NPP enhancement over northern high latitudes. We further report that these differences in NPP responses between the CAS-ESM2 and MME resulted from their different behaviors in simulating NPP trends with modeling time, and are attributed to their different projections of CO_2_-induced climatic anomalies and different climate sensitivities. These results are favorable for understanding and further improving the performance of the CAS-ESM2 in projecting the terrestrial carbon cycle, and point towards a need for greater understanding and improvements for both physical climatic processes and the terrestrial carbon cycle.

## 1. Introduction

Terrestrial net primary production (NPP) is the difference between gross primary production (GPP) and the total carbon gain by photosynthesis, and plant autotrophic respiration (Ra) and the loss of carbon to produce energy for growth and maintenance. NPP makes pivotal contributions to the Earth system by transferring solar energy into organic materials. Changes in NPP are tightly connected with atmospheric CO_2_ concentrations and climate [1]. Understanding these connections and underlying mechanisms is an intense topic of climate sciences.

The manner in which terrestrial NPP responds to increasing atmospheric CO_2_ concentrations and CO_2_-induced climate change comprises a net feedback of GPP and Ra, and is co-controlled by multiple drivers. Much evidence has been obtained to show that terrestrial carbon uptake is enhanced because increasing atmospheric CO_2_ stimulates photosynthetic activity [2,3,4]. For example, Campbell et al. (2017) estimated an increase of 31 ± 5% in global terrestrial GPP during the twentieth century as a result of the increasing CO_2_ [3], and [2] reported an increase of 2.8 ± 1.50% in global terrestrial NPP, from 1982 to 2011, because of elevated CO_2_ [2]. Some observations, however, reveal lower sensitivities of terrestrial production to elevated CO_2_, such as [5] that reported no significant GPP change in a semi-arid grassland in response to elevated CO_2_. Moreover, CO_2_-induced climate change, nutrients availability, and ecosystem structures can also have crucial effects on processes of photosynthesis, leading to more uncertainties in the processes. Higher temperatures caused by elevated CO_2_ can suppress the CO_2_ fertilization effects and negatively influence net ecosystem production [4,6]. Available nutrients can also exert limitations on CO_2_ stimulation [7,8]. Additionally, rapidly rising CO_2_ levels may have less stimulation on vegetation carbon gain than we expected because of the saturating effect [6,9], since the net photosynthetic rate has a nonlinear relationship with CO_2_ concentration [10]. Such evidence illustrates that the response of terrestrial NPP to the rising atmospheric CO_2_ is still complex and uncertain, and that climate change and these associated complexities and uncertainties need more investigation for an accurate understanding of climate and environment change.

Earth system models (ESMs) are a common tool for investigating the responses on a global or regional scale. ESMs have been successively improved in their representation of physical climate systems and terrestrial biogeochemical processes; therefore, they are fundamentally trustworthy to simulate the terrestrial carbon cycle and its interactions with climate [11,12]. The Coupled Model Intercomparison Project (CMIP) has proposed standard experimental protocols for model participants [13,14]. The abruptly quadrupled CO_2_ simulation (abrupt-4 × CO_2_) is a protocol in which atmospheric CO_2_ concentration is fixed as four times that in the pre-industrial simulation (piControl). The fundamental objective of the abrupt-4 × CO_2_ protocol is to project how much the Earth warms in response to increases in greenhouse gases. One common way is to estimate this is with equilibrium climate sensitivities (ECS), which is defined as global surface temperature change when CO_2_ concentration is doubled [15]. ECS ranged from 1.5 to 4.5 K for CMIP5 models, and from 1.8 to 5.6 K for CMIP6 models. Moreover, the protocol allows for an examination of the overall response of terrestrial NPP to the elevated CO_2_ concentration. Studies based on the abrupt-4 × CO_2_ reported that terrestrial NPP is broadly enhanced due to CO_2_ fertilization effects [16]. These protocols and reports provide a common framework and a valuable reference for assessing terrestrial carbon responses to elevated CO_2_.

However, many uncertainties remain in the modeling projections of the terrestrial carbon cycle. A previous study showed that NPP enhancements in abrupt-4 × CO_2_ relative to piControl diverge among the eight CMIP5 ESMs, ranging between 10.8 PgC year^−1^ and 121.6 PgC year^−1^ [16]. The magnitudes of carbon–concentration feedback (mean ± standard deviation = 0.97 ± 0.40 PgC ppm^−1^) and carbon–climate feedback (−45.1 ± 50.6 PgC °C^−1^) over land also show relatively large inter-model spread (one standard deviation) compared with the multi-model mean, and are more uncertain than those over ocean (0.79 ± 0.07 PgC ppm^−1^, −17.2 ± 5.0 PgC °C^−1^); this illustrates divergent behaviors of the 11 CMIP6 ESMs in projecting terrestrial carbon cycles [1]. Uncertainties in the performance of ESMs make it difficult to accurately project future climate change.

These uncertainties result from many sources. Firstly, differences in model structures and parameterizations are a key source. Based on the 12 CMIP5 ESMs, a previous study reported that model structure is responsible for 80% of the uncertainty in projecting terrestrial carbon uptake, overwhelming the roles of emission scenarios and internal variability [17]. Models with nutrient limitations, particularly nitrogen and phosphorus, are generally characterized with weaker carbon–concentration feedback and carbon–climate feedback than models without [1,18]. Great divergence in terrestrial carbon responses can also occur among models with nitrogen limitations as a result of differing parameterizations of key nitrogen cycle processes [19]. Secondly, different climate sensitivities of ESMs to CO_2_ increases can lead to uncertainties in terrestrial carbon responses via carbon–climate feedbacks. ECS based on CMIP6 models range between 1.8 K and 5.6 K, higher than the values (1.5–4.5 K) based on CMIP5 [20]. This increase means persistent uncertainties remain in the projected response of global surface temperature to elevated CO_2_, and consequently in that of the terrestrial carbon cycle. These sources of uncertainties in model projections of the terrestrial carbon cycle show there is an urgent need for systematic assessments and significant improvements for ESMs.

The Chinese Academy of Sciences (CAS) Earth System Model version 2 (CAS-ESM2) has finished the proposed CMIP6 standard experiments [21]. This study used the results of the two experiments, abrupt-4 × CO_2_ and piControl, of the CAS-ESM2, and compared them with those of the CMIP6 models. We focused on assessing the NPP responses of the CAS-ESM2 to elevated CO_2_, and on detecting the underlying causes of the differences with CMIP6 models. The assessments and detections are favorable for understanding the behavior of the CAS-ESM2 deeply, and for further improving the CAS-ESM2 in projecting the terrestrial carbon cycle. A detailed model description, datasets, and methods are in Section 2. Section 3 reports the comparisons of climatic anomalies, NPP anomalies, and the drivers between the CAS-ESM2 and CMIP6 models. A further discussion and perspective is given in Section 4, which is followed by Section 5 that summarizes this research.

## 2. Model Description, Datasets, and Methods

### 2.1. Model Description

The CAS-ESM2 is an Earth system model that was developed by the CAS. The model consists of several components, such as the Atmospheric General Circulation Model (IAP AGCM version 5) [22], the Ocean Model (LICOM version 2) [23], the Common Land Model (CoLM) [24], and the Dynamic Global Vegetation Model (IAP-DGVM) [25,26]. The CAS-ESM2 was built to simulate complex interactions between physical climate systems and the carbon cycle. Detailed information and the performance of the CAS-ESM2 in CMIP6 standard experiments can be found in [21].

NPP in CoLM is calculated as the difference between the simulated GPP and Ra. The calculation occurs in each time step at the level of the plant functional types. An increase in CO_2_ concentration can stimulate vegetation photosynthesis by elevating the internal leaf CO_2_ concentration, and can also enhance water use efficiency by changing leaf stomata. Thus, NPP increases with CO_2_ concentration, and is also influenced by climatic conditions such as temperature, water availability, and photosynthetically active radiation (PAR) [24]. The absorbed PAR is calculated following the method of dividing canopy into sunlit and shaded leaves. Both the sunlit and shaded leaf area indices are associated with leaf area index (LAI), which can be treated in two ways in CoLM. One is to update LAI at each daily time step via the phenology module when dynamic global vegetation model is active. In this way, the climate affects LAI, and changes in LAI in turn regulate the climate. The other treatment of LAI is to read it as an input dataset when the dynamic global vegetation model is inactive. The dataset is based on climatological observations at a monthly resolution. In this way, seasonal variations in LAI can regulate the climate, but there is no response of LAI to climate changes. The two experiments (abrupt-4 × CO_2_ and piControl) of the CAS-ESM2 used in this study treated the LAI following the second way.

### 2.2. Datasets and Methods

Following CMIP6 protocols, we conducted a 500-year piControl simulation and a 150-year abrupt-4 × CO_2_ simulation [21]. Both simulations were conducted at a spatial resolution of 128 × 256, and at a monthly resolution. This research focused on an analysis of variables that included NPP, surface air temperature (TAS), and precipitation (Pre). As a comparison, we downloaded monthly GPP, Ra, TAS, and Pre from CMIP6 models, and treated NPP as the difference between GPP and Ra. On the basis of data availability, we chose 22 CMIP6 models (Table S1) and applied their last 30-year outputs of the piControl simulation, and the first 149-year outputs of the abrupt-4 × CO_2_ simulation. All of the outputs were interpolated into the coarsest spatial resolution of 64 × 128 among the 22 CMIP6 models and in the CAS-ESM2. The responses of annual total NPP, and the differences in TAS and Pre, were all represented as the averaged anomalies for the last 30 years of the 150 years in the abrupt-4 × CO_2_ simulation, relative to the 30-year averages in piControl. To detect the role of TAS and Pre in driving NPP, we used partial correlation and assessed the significance via Student’s *t*-test.

## 3. Results

### 3.1. Climate Anomalies

**Figure 1:**
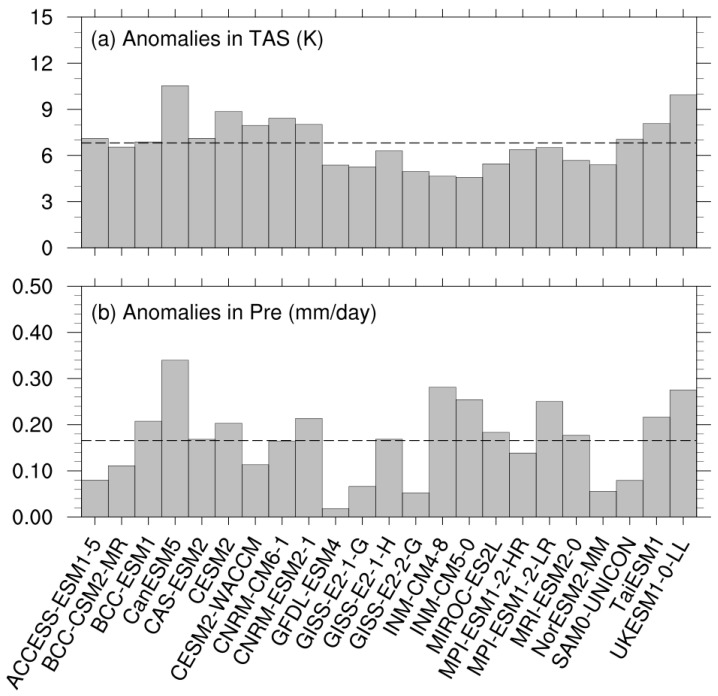
Global averaged anomalies in (**a**) surface air temperature (TAS, units: K) and (**b**) precipitation (Pre, units: mm day^−1^) for the CAS-ESM2 and the 22 CMIP6 models. The dashed lines represent the multi-model ensemble values (MME).

We firstly evaluated the anomalies in surface air temperature and precipitation of the abrupt-4 × CO_2_ simulation, relative to the pre-industrial simulation. Globally, all of the models projected a warmer and wetter climate in the abrupt-4 × CO_2_ than in piControl (Figure 1). The CAS-ESM2 projected an average of 7.11 K warming, slightly higher than the multi-model ensemble mean (hereafter MME; 6.82 K). Of the 22 CMIP6 models, 12 projected less warming than the MME, with INM-CM5-0 showing the weakest amplitude, and the remaining 10 projected more warming than the MME, with CanESM5 showing the strongest amplitude. As for precipitation, the CAS-ESM2 projected a positive anomaly with a value of 0.17 mm day^−1^, which was comparable to the MME (0.17 mm day^−1^). For the 22 CMIP6 models, their one standard deviation of the wetter anomalies was 0.07 mm day^−1^, 41.2% that of the MME, indicating very diverse anomalies in precipitation.

**Figure 2:**
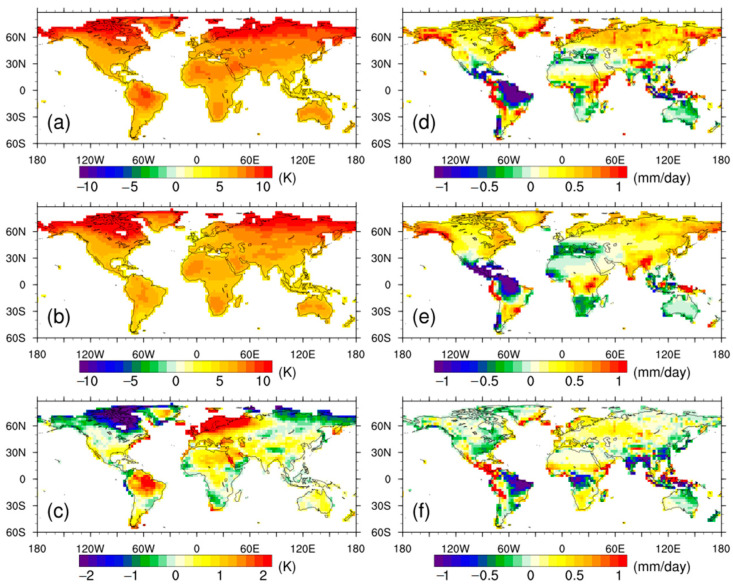
Spatial distribution of anomalies in surface air temperature (TAS, units: K) for (**a**) CAS-ESM2, (**b**) multi-model ensemble mean, and (**c**) their differences. (**d**–**f**) are the same as (**a**–**c**) but for precipitation (Pre, units: mm day^−1^).

Figure 2 shows the spatial pattern of anomalies in surface air temperature and precipitation for the CAS-ESM2 and MME, and compares their differences. For both the CAS-ESM2 and MME, warm anomalies were seen over all land grids, with larger values over northern high latitudes. Their spatial differences showed that the overall stronger warm anomaly of the CAS-ESM2 relative to the MME was mainly contributed by northern Europe and the Amazon (Figure 2c), while northeast Canada and northern Russia were characterized with a weaker warm anomaly for the CAS-ESM2, compared to that for the MME. The spatial pattern of precipitation anomalies showed large regional differences. For the CAS-ESM2 and MME, wetter anomalies occurred over 81.2% and 79.5% of land grids, respectively, while the remaining land grids showed drier anomalies. Most of the wetter anomalies were seen over northern mid-high latitudes, but central America and the Amazon region showed the largest drier anomalies. In comparison to the MME, the CAS-ESM2 projected stronger wetter anomalies or weaker drier anomalies over 51.9% of land grids, including western Eurasia, northern Africa, and southern Africa; meanwhile, stronger drier anomalies or weaker wetter anomalies were seen over the remaining land grids, including the Amazon, central Africa, and tropical Asia (Figure 2f).

These differences in climate anomalies between the CAS-ESM2 and MME, as well as among the 22 CMIP6 models, illustrate their diverse climatic response to quadrupled CO_2_ forcing. These diversities, together with individual model biological processes, contributed to different model behaviors in their NPP responses.

### 3.2. NPP Anomalies

**Figure 3:**
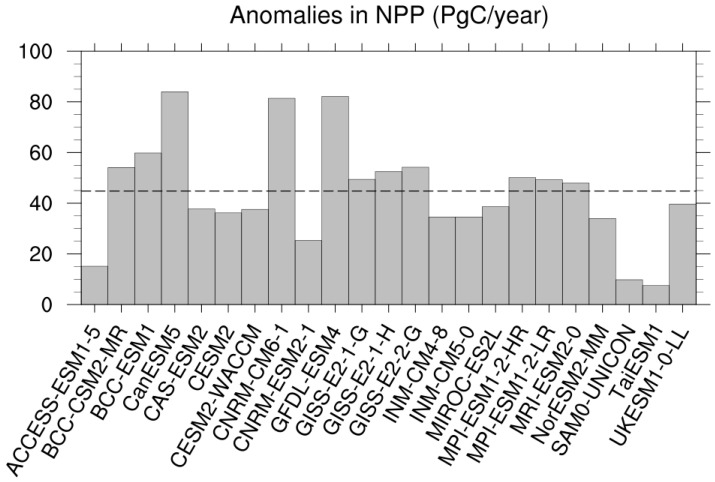
Global annual total NPP anomalies (PgC year^−1^) between simulations of abrupt-4 × CO_2_ and piControl for the CAS-ESM2 and the 22 CMIP6 models. The dashed line represents the multi-model ensemble value (MME).

In response to the quadrupled CO_2_ and the CO_2_-induced climate change, terrestrial NPP showed an overall enhancement for all models at a global scale (Figure 3). The magnitude of the NPP enhancement was very different among the CAS-ESM2 and the 22 CMIP6 models, ranging between 7.56 PgC year^−1^ for TaiESM1 and 83.95 PgC year^−1^ for CanESM5. The CAS-ESM2 projected an enhanced NPP by a value of 37.72 PgC year^−1^, which was slightly (7.06 PgC year^−1^) less than that for the MME. These positive anomalies in NPP for all models are because of the enhancement in GPP which overwhelms that in autotrophic respiration (Figure S1), reflecting the dominant effects of CO_2_ fertilization.

**Figure 4:**
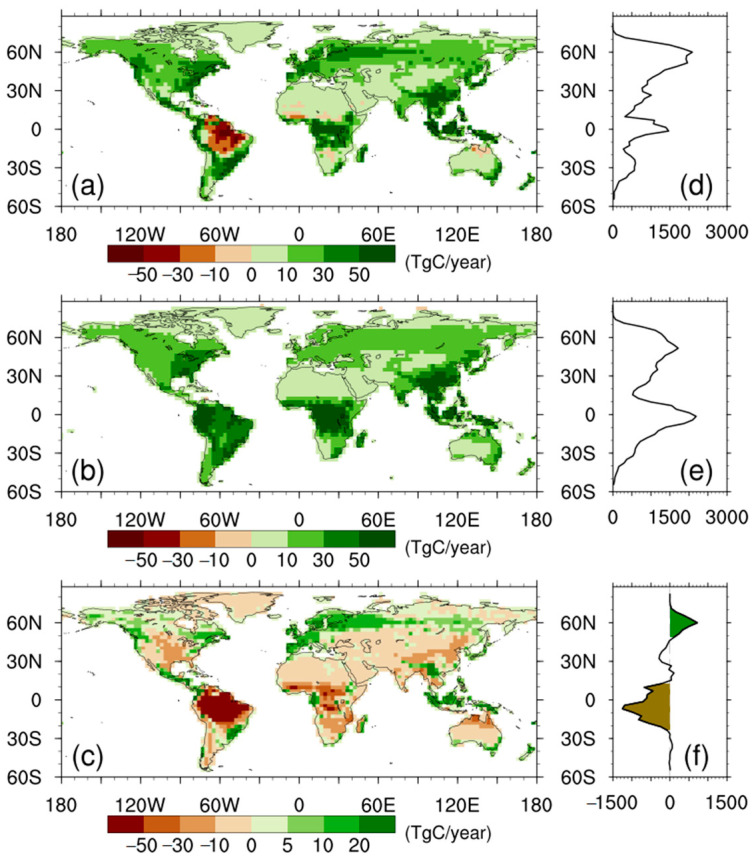
Spatial distribution of NPP anomalies for (**a**) the CAS-ESM2, (**b**) multi-model ensemble, and (**c**) the differences. (**d**–**f**) correspond to the zonal averages. All units are TgC year^−1^.

Figure 4 shows spatial distribution of NPP anomalies for the CAS-ESM2, MME, and their differences. The positive anomalies in NPP for the CAS-ESM2 (Figure 4a) occurred in 92% of land grids, and are mainly attributed to the highly productive regions, including boreal evergreen forests and tropical forests, except for the Amazon region which was characterized with large negative anomalies. For the MME (Figure 4b), the enhanced NPP occurred in almost all land grids, and the mentioned regions with high production, also made larger contributions to the positive anomalies in NPP. Being different from the CAS-ESM2, the MME projected positive NPP anomalies over Amazon forests, even though the enhancement was weaker than that over forests of tropical Africa and tropical Asia. The spatial distribution of differences between the CAS-ESM2 and MME (Figure 4c) shows that weaker NPP enhancement of the CAS-ESM2 relative to MME is seen over 53% of land grids, and was mainly contributed by tropical regions (Figure 4f), including the Amazon and Africa. In reverse, northern high latitudes (i.e., Europe) were characterized with stronger NPP enhancement for the CAS-ESM2 relative to the MME (Figure 4f).

**Figure 5:**
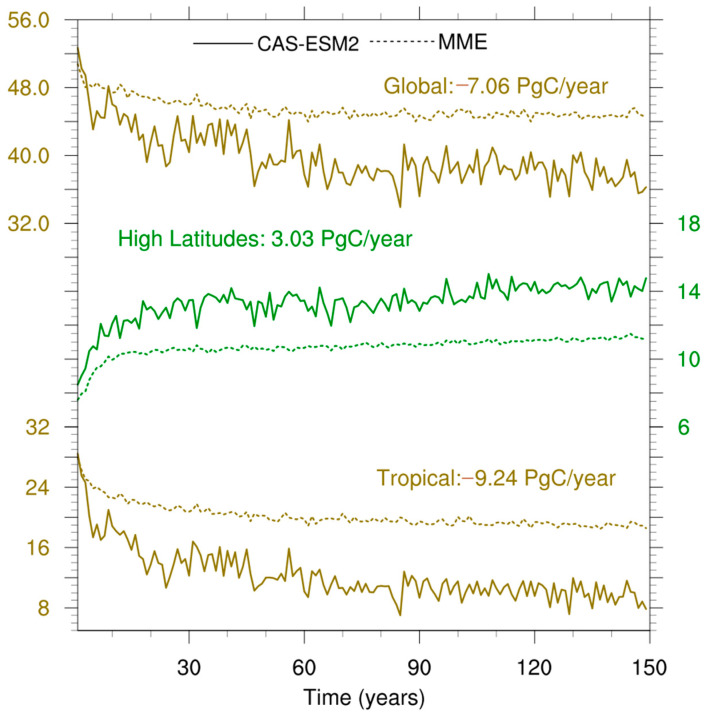
Time evolution of NPP anomalies (units: PgC year^−1^) for the CAS-ESM2 (solid lines) and multi-model ensemble (MME, dashed lines) over the globe (top lines), northern high latitudes (middle lines), and tropics (bottom lines). The numbers represent differences in NPP anomalies between the CAS-ESM2 and MME for the last 30 years.

Figure 5 compares the time evolution of NPP anomalies for the CAS-ESM2 and MME over the globe and two regions colored in Figure 4f. The two regions, northern high latitudes and the tropics, represent regional characteristics with stronger and weaker enhanced NPP of the CAS-ESM2 relative to the MME, respectively. At a global scale, both the CAS-ESM2 and MME projected a decreased trend in NPP anomalies with modeling time, but the CAS-ESM2 showed a steeper one than MME. The difference between the two slopes is the main cause of the overall 7.06 PgC year^−1^, less the NPP enhancement of the CAS-ESM2 relative to the MME for the last 30 modeling years, since their difference in NPP anomalies was slight at the beginning time. The tropics show a similar characteristic as the globe, leading to the 9.24 PgC year^−1^, with less NPP enhancement of the CAS-ESM2 than the MME. Oppositely, northern high latitudes were characterized with an increased trend in NPP anomalies with modeling time, for both the CAS-ESM2 and the MME. Compared to the MME, the CAS-ESM2 simulated 3.03 PgC year^−1^ more NPP enhancement because of its steeper increasing trend in NPP anomaly. 

**Figure 6:**
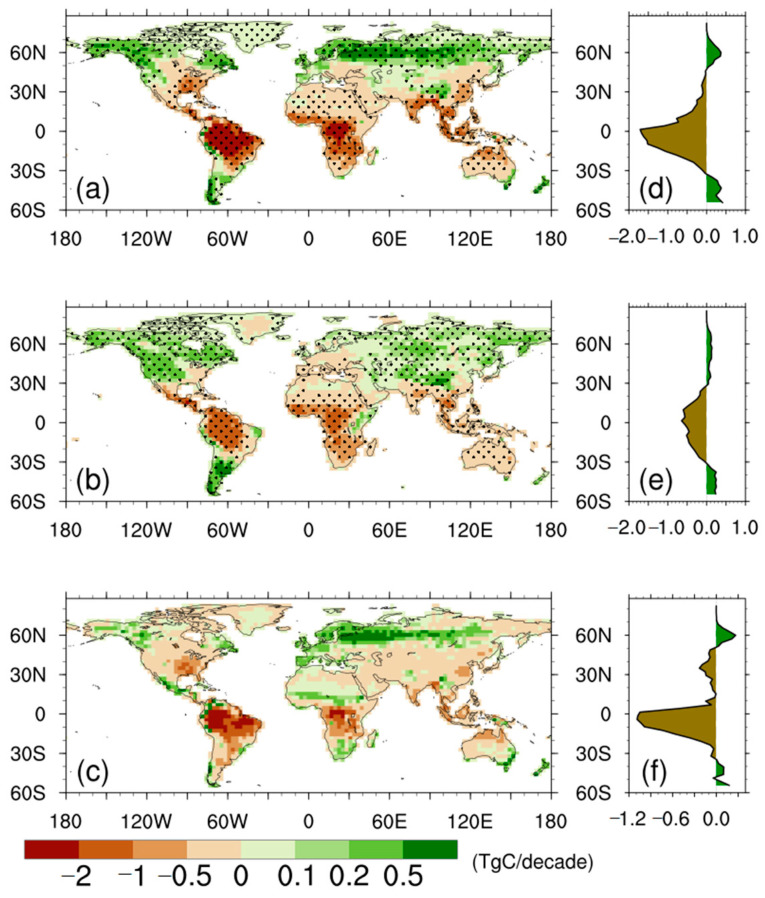
Spatial distribution of trends in NPP anomalies for (**a**) the CAS-ESM2, (**b**) multi-model ensemble, and (**c**) the differences. The stippled regions represent regions with statistical significance (*p* < 0.001). (**d**–**f**) correspond to the zonal averages. All units are TgC decade^−1^.

We further plotted the spatial pattern of the trends in NPP anomalies for the CAS-ESM2 and MME, as well as for their differences (Figure 6). Both the CAS-ESM2 and MME showed overall negative trends in NPP anomalies over tropical ecosystems, while northern mid-high latitudes were characterized with overall positive trends in NPP anomalies. The spatial differences in their trends in NPP anomalies show that the CAS-ESM2 simulated an overall stronger negative trend over tropical ecosystems, but an overall stronger positive trend over northern high latitudes, in comparison with the MME (Figure 6f). Broadly, the spatial pattern of the difference in trends in NPP anomalies (Figure 6c) is consistent with that of the differences in NPP anomalies (Figure 4c). This consistency, together with the results shown in Figure 5, confirm that the differences in NPP anomalies between the CAS-ESM2 and MME for the last 30 modeling years are mainly caused by their different behaviors in simulating NPP trends with modeling time.

### 3.3. Drivers of the Differences in Trends of NPP Anomalies

**Figure 7:**
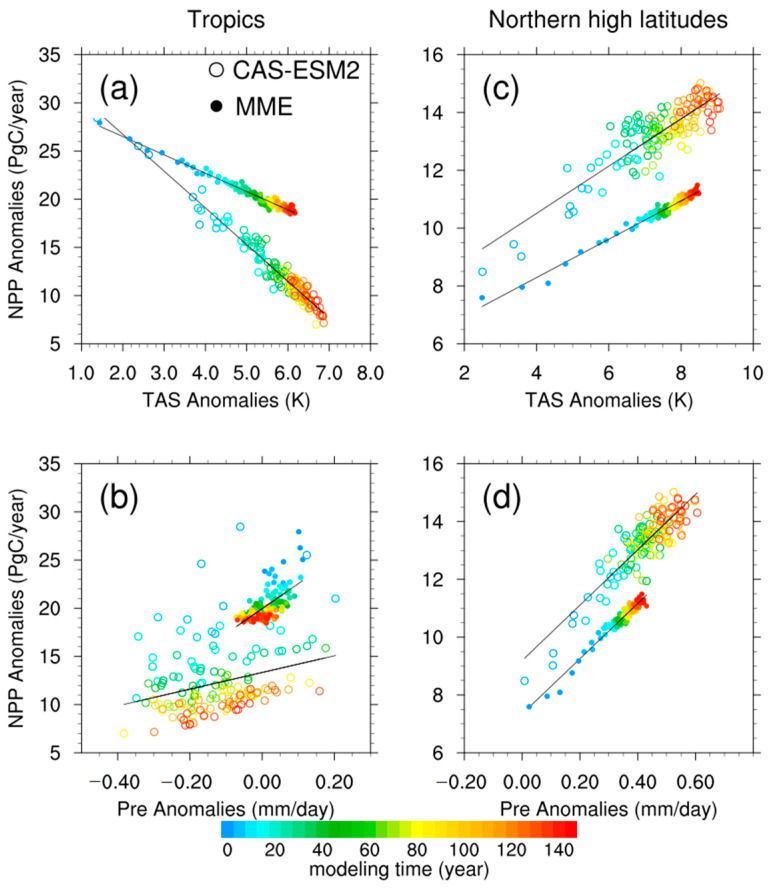
Scatter plots of NPP anomalies (PgC year^−1^) with (**a**) surface air temperature anomalies (TAS; K) and (**b**) precipitation anomalies (Pre; mm day^−1^) over tropics for the CAS-ESM2 (circled dots) and multi-model ensemble (MME; solid dots). Each dot represents a value in each year of the 149-year simulation, and their colors change from cool to warm ones with modeling time. The lines represent their linear regression relationships. (**c**,**d**) are the same as (**a**,**b**) but for northern high latitudes.

Many published studies and our previous research reported on the crucial role of CO_2_-induced climate change in regulating the terrestrial carbon cycle [27,28,29]. The CO_2_-induced warmer and drier climatic conditions can lead to a decreasing trend in terrestrial NPP for the tropics, while northern high latitudes are characterized with an increasing trend in terrestrial NPP because of the CO_2_-induced warmer and wetter climate change. Thus, we next focused on how the anomalies in surface air temperature (TAS) and precipitation (Pre) drive the differences in trends of NPP anomalies between the CAS-ESM2 and MME, for both tropical ecosystems and northern high-latitude ecosystems. Figure 7 and Table 1 compare the relationships between NPP anomalies, TAS and Pre over the two ecosystems for the CAS-ESM2 and MME.

Over tropical ecosystems, both the CAS-ESM2 and MME showed significant and negative correlations between NPP anomalies and TAS anomalies, with partial correlation coefficients −0.99 and −0.98, respectively. Meanwhile, the slope of the CAS-ESM2 (−3.81 PgC K^−1^) is steeper than that of MME (−1.90 PgC K^−1^). In other words, a stronger decrease in tropical NPP will occur for the CAS-ESM2 relative to MME, even if the same increase occurs in TAS. Moreover, the anomaly in tropical TAS for the CAS-ESM2 (6.49 K) is larger than that for MME (6.02 K). These differences in TAS anomalies and the slopes contribute to the stronger negative trend in tropical NPP and the consequently weaker NPP enhancement for the CAS-ESM2, in comparison with the MME. On the other hand, tropical NPP anomalies were significantly and positively correlated with precipitation anomalies, and the partial correlation coefficients were 0.72 and 0.47 for the CAS-ESM2 and MME, respectively. Compared to the MME, even though the CAS-ESM2 shows the gentler slope, the CAS-ESM2 projects a stronger decrease in precipitation, resulting in a stronger decrease in NPP for the CAS-ESM2. Overall, the CAS-ESM2, relative to MME, projects a warmer and drier tropical climate, and its tropical NPP is more sensitive to changes in surface air temperature, which together lead to the relatively weak NPP enhancement.

Over northern high-latitude ecosystems, both the CAS-ESM2 and MME showed significant and positive correlations between NPP anomalies and TAS anomalies (Table 1). Correspondingly, the slopes are 0.82 PgC K^−1^ and 0.66 PgC K^−1^, and the TAS anomalies are 8.48 K and 8.31 K, respectively. This slightly steeper slope and warmer climate of the CAS-ESM2 than those of the MME contribute to its stronger NPP enhancement. On the other hand, the CAS-ESM2 also showed significant and positive correlations between NPP anomalies and precipitation anomalies, while there were no significant correlations for the MME. Compared to the MME, the CAS-ESM2 is also characterized with a slightly steeper slope between NPP anomalies and precipitation anomalies, and with a wetter climate, which further contribute to its relatively strong NPP enhancement. Therefore, for northern high-latitude ecosystems, the 3.03 PgC year^−1^ additional NPP enhancement of the CAS-ESM2 is caused by its warmer and wetter climate and the correspondingly larger sensitivity to these climate changes, in comparison with the MME.

**Figure 8:**
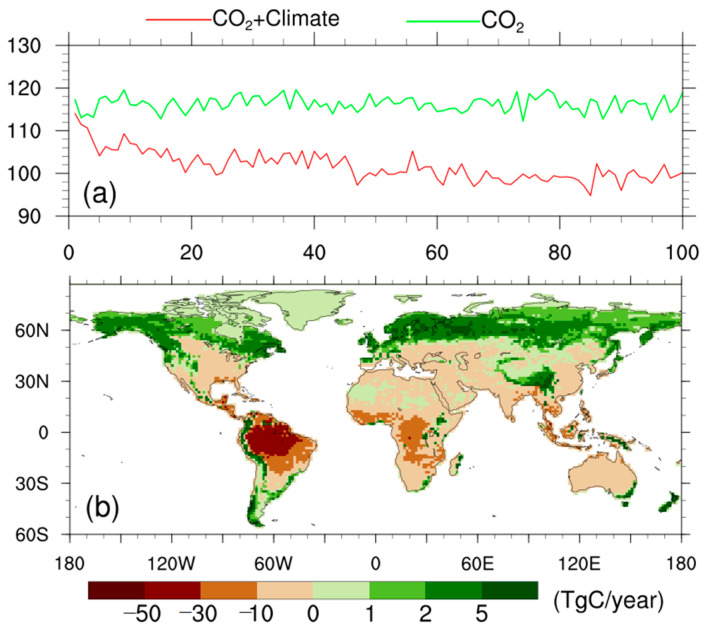
(**a**) Time evolutions of NPP (units: PgC year^−1^) for the biogeochemically coupled simulation (green line) and the fully coupled abrupt-4 × CO_2_ simulation (red line). (**b**) Spatial distribution of the differences in NPP (TgC year^−1^) between the fully coupled abrupt-4 × CO_2_ simulation and the biogeochemically coupled simulation for the last 20 years.

The results attribute the differences to their different behaviors in projecting the associated climatic anomaly, and to different sensitivities to this climatic anomaly. However, in the abrupt-4 × CO_2_ simulation, the CO_2_ fertilization effects change with temperature and water availability; thus, there are interactions with the climatic effects at each time step. To isolate these interactive feedbacks, we further used the CAS-ESM2 to conduct a simulation (biogeochemically coupled simulation), in which the quadrupled CO_2_ affects the terrestrial carbon cycle but not the climate. The differences between the default fully coupled abrupt-4 × CO_2_ simulation and the biogeochemically coupled simulation reflect the climatic effects, as shown in Figure 8.

The green and red lines in Figure 8a represent time evolutions of NPP for the biogeochemically coupled simulation and the fully coupled abrupt-4 × CO_2_ simulation, respectively. Clearly, there is no significant decrease in NPP for the biogeochemically coupled simulation, while the fully coupled abrupt-4 × CO_2_ simulation shows a significant decrease in NPP. This difference leads to an average of 16.7 PgC year^−1^ less NPP for the fully coupled abrupt-4 × CO_2_ simulation relative to the biogeochemically coupled simulation for the last 20 modeling years. The corresponding spatial distribution of the averaged difference shows that the decreased NPP mainly occurs over the tropics, with larger contributions from the Amazon and Africa; meanwhile, northern high latitudes and the Tibet Plateau show slight increases in NPP (Figure 8b). This evidence isolates the climatic effects, and confirms that they are attributable to the NPP decrease for the CAS-ESM2 in the fully coupled abrupt-4 × CO_2_ simulation with modeling time.

## 4. Discussion: Uncertainties and Perspective

**Figure 9:**
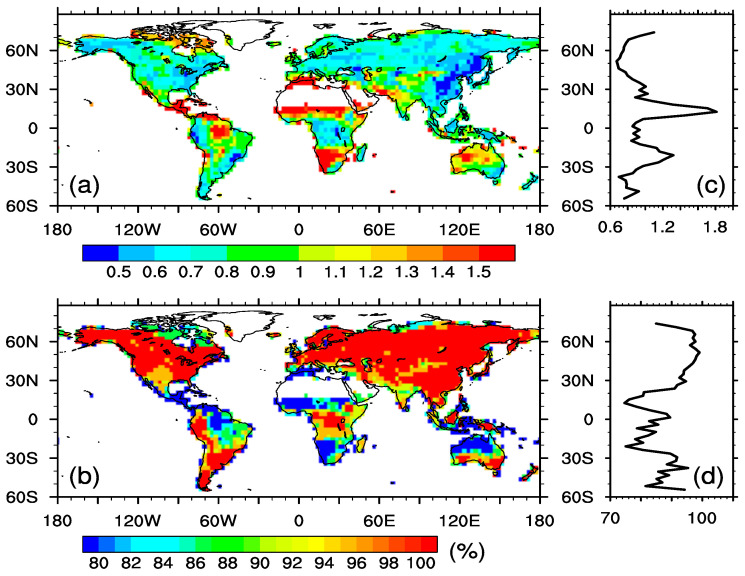
(**a**) shows one standard deviation of overall NPP anomalies among the 23 models. The values are standardized by the multi-model ensemble mean NPP anomaly. (**b**) is the percentage (%) of models that show the same direction as that of the ensemble in overall NPP anomalies. (**c**,**d**) correspond to zonal averages.

This study compared NPP response to abruptly quadrupled CO_2_ and the associated climate change between the CAS-ESM2 and the 22 CMIP6 models. The different NPP responses between the CAS-ESM2 and the 22 CMIP6 models reflect uncertainties in model projections of changes in the terrestrial carbon cycle. We used one standard deviation among the 23 models to represent the inter-model spread, referred to as the uncertainty in magnitudes of overall NPP responses, and standardized the inter-model spread by the ensemble mean NPP anomaly to reduce the effects of absolute NPP values (Figure 9a). Clearly, the standardized inter-model spread of overall NPP anomalies was generally larger over the tropics than over extratropical ecosystems, especially over the Amazon, West Africa, South Africa, and northern Australia. These regions were characterized with opposite NPP responses among the 23 models (Figure S2), which not only reduced the ensemble mean NPP anomaly, but also enhanced inter-model spread, leading to larger standardized inter-model spread. Furthermore, we defined a consistent degree as a percentage of models that showed the same signal in NPP response (positive or negative) as that of the ensemble NPP, and used it to represent uncertainties in the directions of overall NPP anomalies (Figure 9b). The results show lower consistency over the tropics, especially in regions with larger standardized inter-model spread, while extratropical ecosystems broadly show 100% consistency. Overall, these features reveal that the NPP responses were relatively more uncertain over tropical ecosystems than over extratropical ecosystems, in both magnitudes and directions.

These uncertain behaviors come from many sources. Our results emphasized differences in the CO_2_-induced climatic effects, including different associated climatic anomalies and different climate sensitivities. Many previous studies also reported on the crucial effects of model structures and parameterizations on the terrestrial carbon cycle [1,17,18]. For example, Arora et al. (2020) showed that models with or without a representation of nitrogen limitation are different in magnitudes of terrestrial carbon responses to the elevated CO_2_ and the associated climate changes [1]. Similarly, this study reports that models with carbon–nitrogen interactions showed relatively weak NPP increases. The three smallest magnitudes of NPP increase among the 23 models, 15.42 PgC year^−1^, 9.75 PgC year^−1^, and 7.76 PgC year^−1^, were simulated by the ACCESS-ESM1-5, SAM0-UNICON, and TaiESM1 models, respectively. All of them are interactive with nitrogen limitations on the carbon cycle [1]. In contrast, models (CanESM5, CNRM-CM6-1, and GFDL-ESM4) that lacked nitrogen limitations projected the three greatest magnitudes of NPP increase among the 23 models, 82.36 PgC year^−1^, 80.55 PgC year^−1^, and 82.43 PgC year^−1^, respectively. These differences indicate that more efforts are needed to understand and model terrestrial carbon cycle processes accurately.

**Figure 10:**
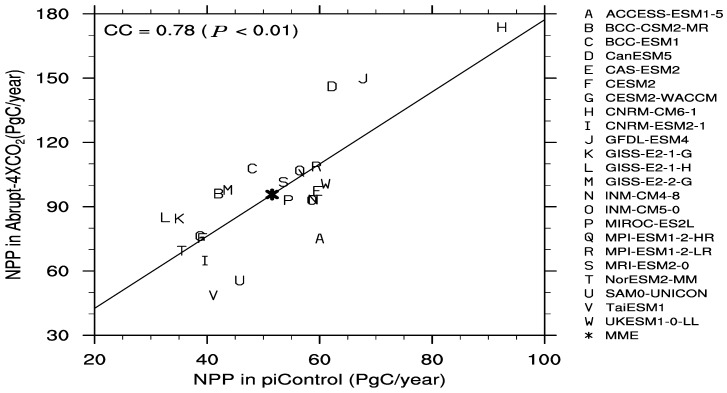
Scatter plot of annual total NPP (PgC year^−1^) in pre-industrial and abrupt-4 × CO_2_ simulations for the 23 models. The line represents their linear regression relationship.

Even though many uncertainties remain, the multi-model ensemble results provide a valuable opportunity to estimate possible changes in the terrestrial carbon cycle in response to quadrupled CO_2_ and the associated climate change. Figure 10 shows the relationship between the simulated global NPP in piControl (NPP_PI_) and that in the abrupt-4 × CO_2_ simulation (NPP_4CO2_) among the 23 CMIP6 models. It is interesting to note that NPP_4CO2_ is significantly correlated with NPP_PI_, and the correlation coefficient is 0.78 (*p* < 0.01). This means that the magnitude of NPP in the abrupt-4 × CO_2_ simulation is associated with the pre-industrial NPP value. In other words, a model can project a more reliable NPP_4CO2_ if it can reproduce a more accurate NPP_PI_. This research reported that the multi-model ensemble NPP_PI_ was 51.55 PgC year^−1^, which is slightly less than the estimation based on the Moderate Resolution Imaging Spectroradiometer (MODIS) product, 54.00 PgC year^−1^ [14]. This is relatively reasonable, in consideration of the different climatic conditions between the pre-industrial era and the time when MODIS estimation was applied. Thus, the multi-model ensemble provided a reasonable and valuable NPP estimation (95.71 PgC year^−1^) in the abrupt-4 × CO_2_ simulation. For the CAS-ESM2, the NPP_4CO2_ value (97.38 PgC year^−1^) was also reasonable according to the significant relationship between NPP_4CO2_ and NPP_PI_.

## 5. Conclusions

This study applied the CMIP6 standard pre-industrial experiment (piControl) and the abruptly quadrupled CO_2_ experiment (abrupt-4 × CO_2_) to investigate terrestrial NPP responses to elevated CO_2_ and CO_2_-induced climate change. The research focused on projections of the CAS-ESM2, and on the underlying causes of its differences with the 22 CMIP6 models. We firstly showed climatic anomalies that were characterized with a warmer and wetter climate in the abrupt-4 × CO_2_ than in piControl for all models. The CAS-ESM2 projected a slightly (0.29 K) stronger warming than the MME, which was mainly contributed by northern Europe and the Amazon, while their precipitation anomalies were comparable. Then, we reported an overall enhanced NPP occurring for all models at a global scale, reflecting the dominant effects of CO_2_ fertilization. The CAS-ESM2 projected a 37.72 PgC year^−1^ NPP enhancement, slightly (7.06 PgC year^−1^, 16%) less than that of the MME. This weaker NPP enhancement of the CAS-ESM2 relative to the MME was mainly attributed to tropical regions, including the Amazon and Africa, while northern high latitudes showed relatively strong NPP enhancement. Further analysis investigated the causes of these differences in NPP anomalies between the CAS-ESM2 and MME. This study highlights the role of their different CO_2_-induced climatic anomalies and different sensitivities to these climatic anomalies, while their sensitivities of photosynthetic capacity to CO_2_ can also be crucial underlying causes.

Our results reported the estimation of NPP responses to the quadrupled CO_2_ in the CAS-ESM2, and emphasized the role of CO_2_-induced climatic effects in inducing uncertainties in projecting the terrestrial carbon cycle. The research is very important for the CAS-ESM2 group and other modeling groups. Firstly, the results provide valuable information about the overall performance of the CAS-ESM2 in projecting NPP responses to elevated CO_2_, which can serve as a benchmark for modeling groups. Secondly, the vegetation was static and leaf area index also was not changed with climate in this study, which limited the interactions between vegetation and climate. Further research is needed to re-assess these responses by including a dynamic global vegetation model. Thirdly, the results reveal the crucial effects of CO_2_-induced climate changes on terrestrial NPP. The climate sensitivities of models to elevated CO_2_ can be reflected by equilibrium climate sensitivities. Therefore, quantitative assessment of the contributors to the differences in equilibrium climate sensitivities can lead to insights into the differences in NPP responses. Finally, the uncertainty analysis in this study reported hot spots with larger uncertainties in projecting NPP responses, such as the Amazon region and West Africa. This information is pivotal for modeling groups to further improve their ability to simulate the terrestrial carbon cycle.

## Figures and Tables

**Table 1:** Values of the analyzed variables for the CAS-ESM2 and multi-model ensemble (MME) over tropics and northern high latitudes. These variables include anomalies in NPP (units: PgC years^−1^); surface air temperature (TAS; units: K); precipitation (Pre; units: mm day^−1^); partial correlation coefficients between NPP and surface air temperature (PCC_NPP&TAS_Pre_), and those between NPP and precipitation (PCC_NPP&Pre_TAS_); slopes between NPP and surface air temperature (Slope_NPP&TAS_, units: PgC year^−1^/K), and those between NPP and precipitation (Slope_NPP&Pre_, PgC year^−1^/(mm day^−1^)). Asterisks represent values that are statistically significant (*p* < 0.001).

	Tropics		Northern High Latitudes	
	CAS-ESM2	MME	CAS-ESM2	MME
NPP Anomalies	9.76	19.00	14.23	11.20
TAS Anomalies	6.49	6.02	8.48	8.31
Pre Anomalies	−0.10	−0.01	0.53	0.40
PCC_NPP&TAS_Pre_	−0.99 *	−0.98 *	0.39 *	0.64 *
PCC_NPP&Pre_TAS_	0.72 *	0.47 *	0.48 *	0.05
Slope_NPP&TAS_	−3.81	−1.90	0.82	0.66
Slope_NPP&Pre_	8.66	25.94	9.58	9.56

## Data Availability

Not applicable.

## Supplementary Materials

The following supporting information can be downloaded at: https://www.mdpi.com/article/10.3390/biology11121693/s1, Figure S1: Global annual total anomalies in (a) GPP and (b) Ra between simulations of abrupt-4 × CO_2_ and piControl for the CAS-ESM2 and the 22 CMIP6 models; Figure S2: Spatial distributions of annual total NPP differences (TgC year−1) between abrupt-4 × CO_2_ and pre-industrial simulation for the 23 models; Table S1: The selected CMIP6 models and corresponding references [21,30,31,32,33,34,35,36,37,38,39,40,41,42,43,44,45,46,47,48,49].
