# Supplementary material for: Response of Terrestrial Net Primary Production to Quadrupled CO2 Forcing: A Comparison between the CAS-ESM2 and CMIP6 Models
Source: Biology (Basel). 2022 Nov 24;11(12):1693. doi: 10.3390/biology11121693 (PMC9774443; doi:10.3390/biology11121693)
Supplement: Supplementary file 1 [file biology-11-01693-s001.zip › biology-1992302-supplementary.pdf]

Supporting Information for

Response of Terrestrial Net Primary Production to Quadrupled CO_2 Forcing: A Comparison between the CAS-ESM2 and CMIP6 Models

Jiawen Zhu[*1,2], Xiaodong Zeng[1,2,3]， Xiaofei Gao[1,3]， He Zhang[1]

[1]International Center for Climate and Environment Sciences,

Institute of Atmospheric Physics, Chinese Academy of Sciences, Beijing 100029, China

[2] Collaborative Innovation Center on Forecast and Evaluation of Meteorological Disasters (CIC-FEMD), Nanjing University of Information Science & Technology, Nanjing 210044, China.

[3] University of Chinese Academy of Sciences, Beijing 100049, China

*Corresponding author: Jiawen Zhu (Email: zhujw@mail.iap.ac.cn)

Contents of this file

Figures S1 to S2

Table S1

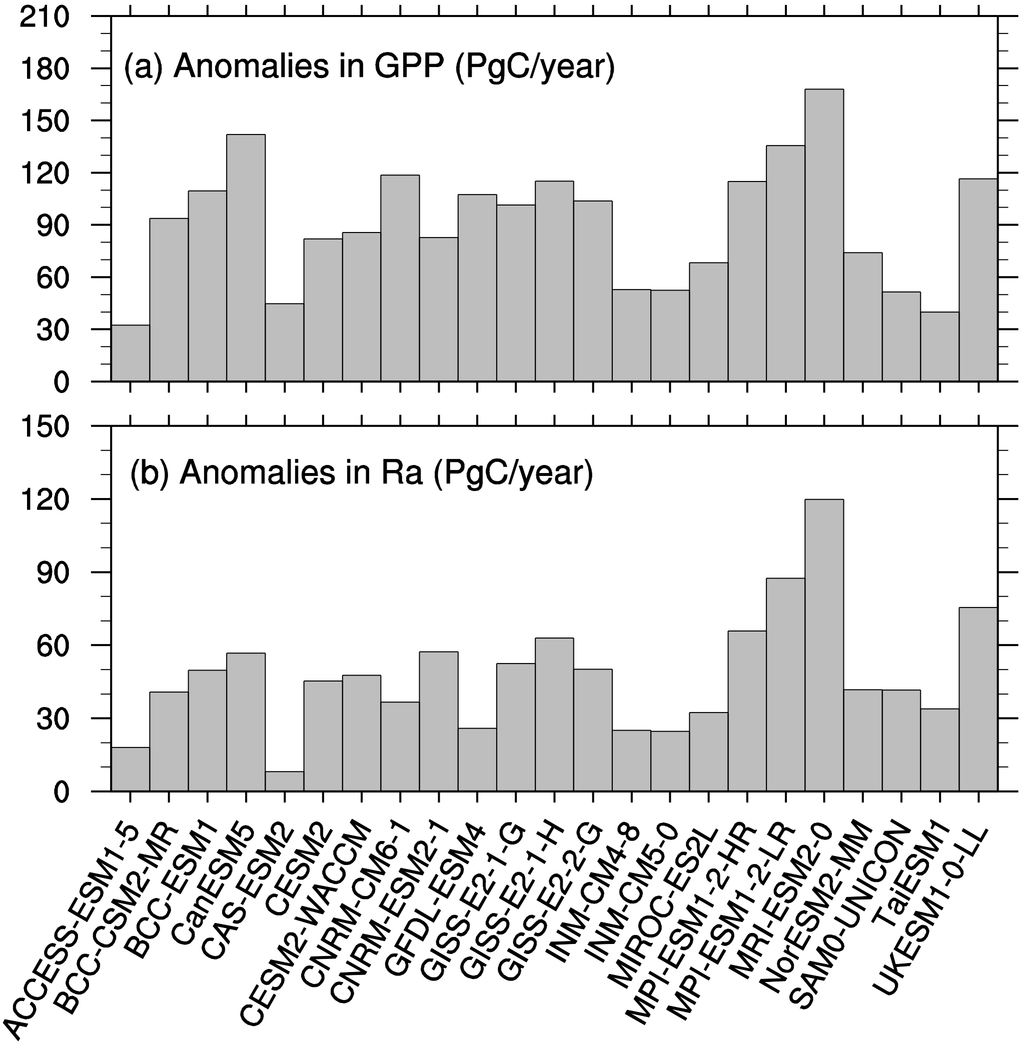

Figure S1. Global annual total anomalies in (a) GPP and (b) Ra between simulations of abrupt-4×CO_2 and piControl for CAS-ESM2 and the 22 CMIP6 models.

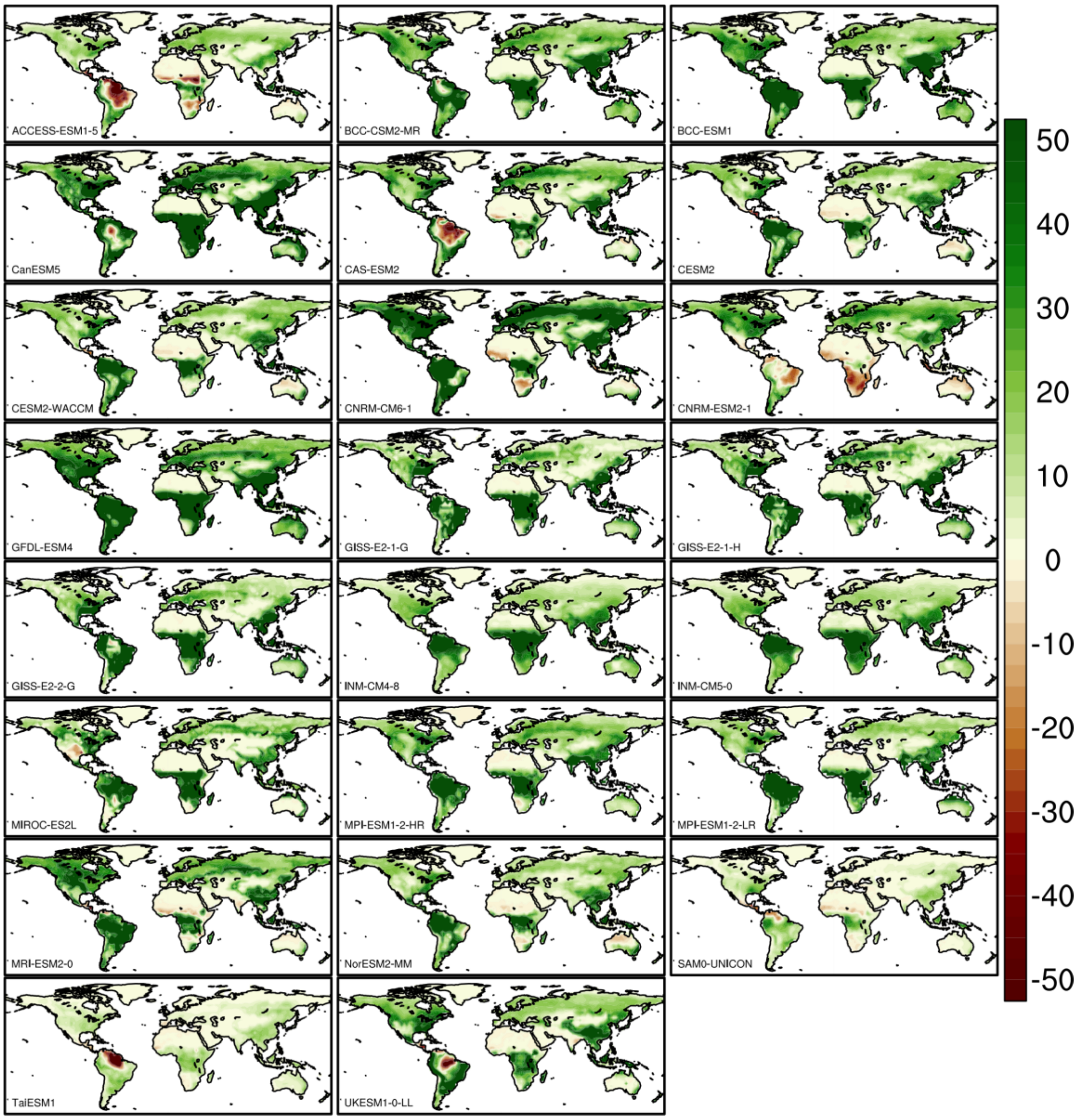

Figure S2. Spatial distributions of annual total NPP differences (TgC year^{-1}) between abrupt-4×CO_2 and pre-industrial simulation for the 23 models.

Table S1. The selected CMIP6 models and corresponding references

Model name	Reference
ACCESS-ESM1-5	[1]
BCC-CSM2-MR	[2]
BCC-ESM1	[3]
CanESM5	[4]
CAS-ESM2	[5]
CESM2	[6]
CESM2-WACCM	[6]
CNRM-CM6-1	[7]
CNRM-ESM2-1	[8]
GFDL-ESM4	[9]
GISS-E2-1-G	[10]
GISS-E2-1-H	[10]
GISS-E2-2-G	[11]
INM-CM4-8	[12]
INM-CM5-0	[13]
MIROC-ES2L	[14]
MPI-ESM1-2-HR	[15]
MPI-ESM1-2-LR	[16]
MRI-ESM2-0	[17]
NorESM2-MM	[18]
SAM0-UNICON	[19]
TaiESM1	[20]
UKESM1-0-LL	[21]

References

1. Ziehn, T.; Chamberlain, M.A.; Law, R.M.; Lenton, A.; Bodman, R.W.; Dix, M.; Stevens, L.; Wang, Y.-P.; Srbinovsky, J. The Australian Earth System Model: ACCESS-ESM1.5. *J. South. Hemisph. Earth Syst. Sci.* **2020**, *70*, 193–214. https://doi.org/10.1071/ES19035.
2. Wu, T.; Lu, Y.; Fang, Y.; Xin, X.; Li, L.; Li, W.; Jie, W.; Zhang, J.; Liu, Y.; Zhang, L.; et al. The Beijing Climate Center Climate System Model (BCC-CSM): The main progress from CMIP5 to CMIP6. *Geosci. Model Dev.* **2019**, *12*, 1573–1600. https://doi.org/10.5194/gmd-12-1573-2019.
3. Wu, T.; Zhang, F.; Zhang, J.; Jie, W.; Zhang, Y.; Wu, F.; Li, L.; Yan, J.; Liu, X.; Lu, X.; et al. Beijing Climate Center Earth System Model version 1 (BCC-ESM1): Model description and evaluation of aerosol simulations. *Geosci. Model Dev.* **2020**, *13*, 977–1005. https://doi.org/10.5194/gmd-13-977-2020.
4. Swart, N.C.; Cole, J.N.S.; Kharin, V.V.; Lazare, M.; Scinocca, J.F.; Gillett, N.P.; Anstey, J.; Arora, V.; Christian, J.R.; Hanna, S.; et al. The Canadian Earth System Model version 5 (CanESM5.0.3). *Geosci. Model Dev.* **2019**, *12*, 4823–4873. https://doi.org/10.5194/gmd-12-4823-2019.
5. Zhang, H.; Zhang, M.H.; Jin, J.B.; Fei, K.C.; Ji, D.Y.; Wu, C.L.; Zhu, J.W.; He, J.X.; Chai, Z.Y.; Xie, J.B.; et al. Description and Climate Simulation Performance of CAS-ESM Version 2. *J. Adv. Model. Earth Syst.* **2020**, *12*, e2020MS002210. https://doi.org/10.1029/2020ms002210.
6. Danabasoglu, G.; Lamarque, J.-F.; Bacmeister, J.; Bailey, D.A.; DuVivier, A.K.; Edwards, J.; Emmons, L.K.; Fasullo, J.; Garcia, R.; Gettelman, A.; et al. The Community Earth System Model Version 2 (CESM2). *J. Adv. Model. Earth Syst.* **2020**, 12, e2019MS001916. https://doi.org/10.1029/2019MS001916.
7. Voldoire, A.; Saint-Martin, D.; Sénési, S.; Decharme, B.; Alias, A.; Chevallier, M.; Colin, J.; Guérémy, J.-F.; Michou, M.; Moine, M.-P.; et al. Evaluation of CMIP6 DECK Experiments With CNRM-CM6-1. *J. Adv. Model. Earth Syst.* **2019**, *11*, 2177–2213. https://doi.org/10.1029/2019MS001683.
8. Séférian, R.; Nabat, P.; Michou, M.; Saint-Martin, D.; Voldoire, A.; Colin, J.; Decharme, B.; Delire, C.; Berthet, S.; Chevallier, M.; et al. Evaluation of CNRM Earth System Model, CNRM-ESM2-1: Role of Earth System Processes in Present-

Day and Future Climate. *J. Adv. Model. Earth Syst.* **2019**, *11*, 4182–4227. https://doi.org/10.1029/2019MS001791.

9. Dunne, J.P.; Horowitz, L.W.; Adcroft, A.J.; Ginoux, P.; Held, I.M.; John, J.G.; Krasting, J.P.; Malyshev, S.; Naik, V.; Paulot, F.; et al. The GFDL Earth System Model Version 4.1 (GFDL-ESM 4.1): Overall Coupled Model Description and Simulation Characteristics. *J. Adv. Model. Earth Syst.* **2020**, 12, e2019MS002015. https://doi.org/10.1029/2019MS002015.
10. Kelley, M.; Schmidt, G.A.; Nazarenko, L.S.; Bauer, S.E.; Ruedy, R.; Russell, G.L.; Ackerman, A.S.; Aleinov, I.; Bauer, M.; Bleck, R.; et al. GISS-E2.1: Configurations and Climatology. *J. Adv. Model. Earth Syst.* **2020**, 12, e2019MS002025. https://doi.org/10.1029/2019MS002025.
11. Rind, D.; Orbe, C.; Jonas, J.; Nazarenko, L.; Zhou, T.; Kelley, M.; Lacis, A.; Shindell, D.; Faluvegi, G.; Romanou, A.; et al. GISS Model E2.2: A Climate Model Optimized for the Middle Atmosphere—Model Structure, Climatology, Variability, and Climate Sensitivity. *J. Geophys. Res.: Atmos.* **2020**, 125, e2019JD032204. https://doi.org/10.1029/2019JD032204.
12. Volodin, E.M.; Mortikov, E.V.; Kostrykin, S.V.; Galin, V.Y.; Lykossov, V.N.; Gritsun, A.S.; Diansky, N.A.; Gusev, A.V.; Iakovlev, N.G.; Shestakova, A.A.; et al. Simulation of the modern climate using the INM-CM48 climate model. *Russ. J. Numer. Anal. Math. Model.* **2018**, *33*, 367–374. https://doi.org/doi:10.1515/rnam-2018-0032.
13. Volodin, E.; Gritsun, A. Simulation of observed climate changes in 1850–2014 with climate model INM-CM5. *Earth Syst. Dynam.* **2018**, *9*, 1235–1242. https://doi.org/10.5194/esd-9-1235-2018.
14. Hajima, T.; Watanabe, M.; Yamamoto, A.; Tatebe, H.; Noguchi, M.A.; Abe, M.; Ohgaito, R.; Ito, A.; Yamazaki, D.; Okajima, H.; et al. Development of the MIROC-ES2L Earth system model and the evaluation of biogeochemical processes and feedbacks. *Geosci. Model Dev.* **2020**, *13*, 2197–2244. https://doi.org/10.5194/gmd-13-2197-2020.
15. Müller, W.A.; Jungclaus, J.H.; Mauritsen, T.; Baehr, J.; Bittner, M.; Budich, R.; Bunzel, F.; Esch, M.; Ghosh, R.; Haak, H.; et al. A Higher-resolution Version of the Max Planck Institute Earth System Model (MPI-ESM1.2-HR). *J. Adv. Model. Earth Syst.* **2018**, *10*, 1383–1413. https://doi.org/10.1029/2017MS001217.

16. Mauritsen, T.; Bader, J.; Becker, T.; Behrens, J.; Bittner, M.; Brokopf, R.; Brovkin, V.; Claussen, M.; Crueger, T.; Esch, M.; et al. Developments in the MPI-M Earth System Model version 1.2 (MPI-ESM1.2) and Its Response to Increasing CO_2. *J. Adv. Model. Earth Syst.* **2019**, *11*, 998–1038. https://doi.org/10.1029/2018MS001400.
17. Yukimoto, S.; Kawai, H.; Koshiro, T.; Oshima, N.; Yoshida, K.; Urakawa, S.; Tsujino, H.; Deushi, M.; Tanaka, T.; Hosaka, M.; et al. The Meteorological Research Institute Earth System Model Version 2.0, MRI-ESM2.0: Description and Basic Evaluation of the Physical Component. *J. Meteorol. Soc. Jpn. Ser. II* **2019**, *97*, 931–965. https://doi.org/10.2151/jmsj.2019-051.
18. Seland, Ø.; Bentsen, M.; Olivié, D.; Toniazzo, T.; Gjermundsen, A.; Graff, L.S.; Debernard, J.B.; Gupta, A.K.; He, Y.C.; Kirkevåg, A.; Schwinger, J.; et al. Overview of the Norwegian Earth System Model (NorESM2) and key climate response of CMIP6 DECK, historical, and scenario simulations. *Geosci. Model Dev.* **2020**, *13*, 6165–6200. https://doi.org/10.5194/gmd-13-6165-2020.
19. Park, S.; Shin, J.; Kim, S.; Oh, E.; Kim, Y. Global Climate Simulated by the Seoul National University Atmosphere Model Version 0 with a Unified Convection Scheme (SAM0-UNICON). *J. Clim.* **2019**, *32*, 2917–2949. https://doi.org/10.1175/jcli-d-18-0796.1.
20. Lee, W.L.; Wang, Y.C.; Shiu, C.J.; Tsai, I.; Tu, C.Y.; Lan, Y.Y.; Chen, J.P.; Pan, H.L.; Hsu, H.H. Taiwan Earth System Model Version 1: Description and evaluation of mean state. *Geosci. Model Dev.* **2020**, *13*, 3887–3904. https://doi.org/10.5194/gmd-13-3887-2020.
21. Sellar, A.A.; Jones, C.G.; Mulcahy, J.P.; Tang, Y.; Yool, A.; Wiltshire, A.; O’Connor, F.M.; Stringer, M.; Hill, R.; Palmieri, J.; et al. UKESM1: Description and Evaluation of the U.K. Earth System Model. *J. Adv. Model. Earth Syst.* **2019**, *11*, 4513–4558. https://doi.org/10.1029/2019MS001739.
